# Targeting IL-1**β** as an immunopreventive and therapeutic modality for *K-ras*–mutant lung cancer

**DOI:** 10.1172/jci.insight.157788

**Published:** 2022-06-08

**Authors:** Bo Yuan, Michael J. Clowers, Walter V. Velasco, Stephen Peng, Qian Peng, Yewen Shi, Marco Ramos-Castaneda, Melody Zarghooni, Shuanying Yang, Rachel L. Babcock, Seon Hee Chang, John V. Heymach, Jianjun Zhang, Edwin J. Ostrin, Stephanie S. Watowich, Humam Kadara, Seyed Javad Moghaddam

**Affiliations:** 1 Department of Pulmonary Medicine, The University of Texas MD Anderson Cancer Center, Houston, Texas, USA.; 2 Department of Pulmonary and Critical Care Medicine, The Second Affiliated Hospital of Xi’an Jiaotong University, Xi’an, China.; 3 The University of Texas MD Anderson Cancer Center, UTHealth Graduate School of Biomedical Sciences, Houston, Texas, USA.; 4 Department of General Internal Medicine,; 5 Department of Head & Neck Surgery,; 6 Department of Immunology,; 7 Department of Thoracic Head & Neck Medical Oncology, and; 8 Department of Translational Molecular Pathology, The University of Texas MD Anderson Cancer Center, Houston, Texas, USA.

**Keywords:** Oncology, Lung cancer, NF-kappaB, Neutrophils

## Abstract

*K-ras*–mutant lung adenocarcinoma (KM-LUAD) is associated with abysmal prognosis and is tightly linked to tumor-promoting inflammation. A human mAb, canakinumab, targeting the proinflammatory cytokine IL-1β, significantly decreased the risk of lung cancer in the Canakinumab Anti-inflammatory Thrombosis Outcomes Study. Interestingly, we found high levels of IL-1β in the lungs of mice with *K-ras*^G12D^–mutant tumors (CC-LR mice). Here, we blocked IL-1β using an anti–IL-1β mAb in cohorts of 6- or 14-week-old CC-LR mice to explore its preventive and therapeutic effect, respectively. IL-1β blockade significantly reduced lung tumor burden, which was associated with reprogramming of the lung microenvironment toward an antitumor phenotype characterized by increased infiltration of cytotoxic CD8^+^ T cells (with high IFN-γ and granzyme B expression but low programmed cell death 1 [PD-1] expression) while suppressing neutrophils and polymorphonuclear (PMN) myeloid-derived suppressor cells. When querying the Cancer Genome Atlas data set, we found positive correlations between *IL1B* expression and infiltration of immunosuppressive PMNs and expression of their chemoattractant, *CXCL1*, and *PDCD1* expressions in patients with KM-LUAD. Our data provide evidence that IL-1β blockade may be a preventive strategy for high-risk individuals and an alternative therapeutic approach in combination with currently available treatments for KM-LUAD.

## Introduction

Lung cancer remains the leading cause of cancer-related mortality, with an estimated 1.8 million deaths (18% of all sites) in 2020 (1). Non–small cell lung cancer (NSCLC) accounts for 80% of lung cancer cases, and lung adenocarcinoma (LUAD) is its predominant histological subtype (2). *K-ras* mutations are the most common oncogenic aberrations in LUAD, especially in lifetime smokers (3), and are characterized by abysmal prognosis and suboptimal response to most forms of systemic and targeted therapies (4). Therefore, strategies to prevent or treat *K-ras*–mutant LUAD (KM-LUAD) in its earliest stages among high-risk individuals such as smokers are urgently needed to reduce the public burden of this fatal disease. This urgent need for new strategies becomes even more significant because of the persistent risk among former smokers and increased rate of diagnosis of early-stage lung cancer with low-dose CT screening in high-risk populations (e.g., heavy smokers with or without chronic obstructive pulmonary disease [COPD]). These advances heavily rely on understanding mechanisms underlying the promotion of early lung lesions and that may constitute ideal targets for personalized chemopreventive strategies.

Many cancers arise in the context of chronic inflammation (5, 6). Inflammation initiated by intrinsic (e.g., oncogene activation) or extrinsic (e.g., infection) factors could promote cancer by inducing the production of various cytokines or chemokines. These secreted factors can then affect malignant cells and different types of stromal, innate, and adaptive immune cells through induction of an inflammatory signaling network and subsequently reprogram the tumor microenvironment (TME) (7, 8). Our group and others have shown that activation of oncogenic *K-ras* in the lung (mouse and human) is associated with intrinsic inflammatory responses composing activation of the NF-κB pathway, increased expression of inflammatory cytokines including IL-6, IL-17A, and IL-22, and subsequent induction of an immunosuppressive lung microenvironment (9–12). We have further shown a functional role for these cytokines in the pathogenesis of KM-LUAD. IL-1β, a potent activator of the NF-κB pathway, is a proinflammatory cytokine belonging to the IL-1 family of proteins that functions mainly upstream to the aforementioned cytokines (13, 14). Elevated levels of IL-1β are associated with tumor initiation, invasiveness, and progression in a variety of malignancies (15–17). Indeed, IL-1β is abundant at tumor sites, including lung tumors; high levels of IL-1β were found in the serum and tissues of patients with lung cancer and correlated with poor prognosis (18–20). In addition, canakinumab, a human mAb targeting IL-1β, was found to significantly decrease the risk for and mortality associated with invasive lung cancer in the Canakinumab Anti-inflammatory Thrombosis Outcomes Study (CANTOS) (21). Despite these insights on the potential implication of IL-1β in lung cancer clinical outcomes, the role of this proinflammatory cytokine in the early pathogenesis of KM-LUAD is poorly understood.

Here, we studied the role of IL-1β and its potential mechanism of action in the pathogenesis of KM-LUAD in mice. We found that IL-1β blockade significantly decreased the lung tumor burden in both preventive and therapeutic settings while modulating the lung microenvironment toward an antitumor phenotype.

## Results

### Immunopreventive targeting of IL-1β decreases tumor burden, tumor cell proliferation, and tumor angiogenesis while increasing tumor cell apoptosis.

**Figure 1:**
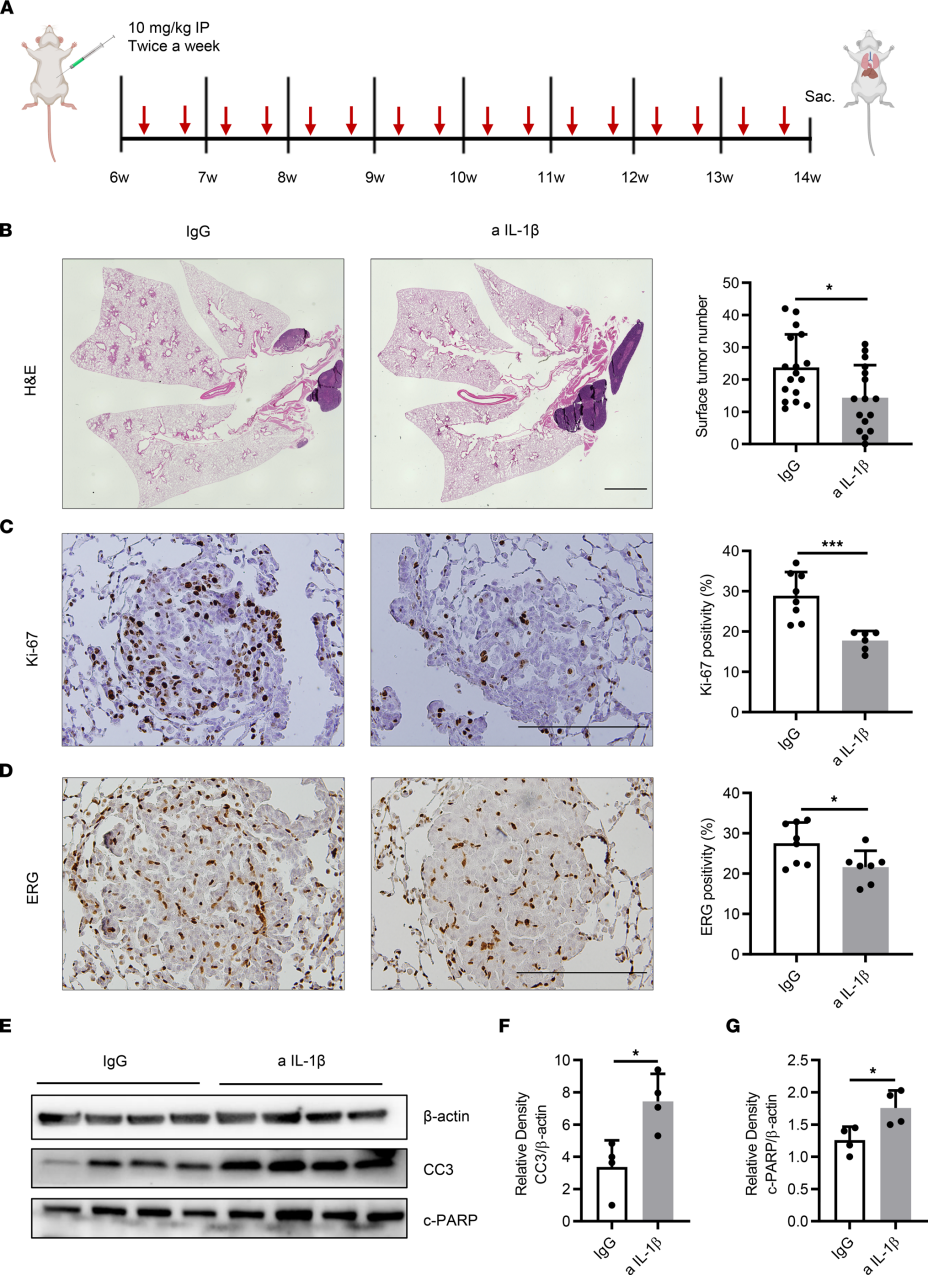
Immunopreventive targeting of IL-1β decreases tumor burden, tumor cell proliferation, and tumor angiogenesis while increasing tumor cell apoptosis. (**A**) Schematic illustration of the experimental plan and procedure. CC-LR mice received IgG control or anti–IL-1β Ab at the age of 6 weeks and were sacrificed at 14 weeks of age. (**B**) Lung-surface tumor number (*n* = 16–17) and photomicrographs of the H&E–stained lung sections in 14-week-old CC-LR mice treated with IgG or anti–IL-1β Ab. Original magnification, ×4. Scale bar: 2 mm. (**C**) Representative photomicrographs of Ki-67–stained sections (original magnification, ×20; scale bar: 200 μm) and quantification of Ki-67 staining presented as the percentage of Ki-67–positive cells (*n* = 6–8). (**D**) Representative photomicrographs of ERG-stained sections (original magnification, ×20; scale bar: 200 μm) and quantification of ERG staining presented as the percentage of ERG-positive cells (*n* = 7–8). (**E**) WB analysis of CC3, c-PARP, and β-actin protein levels in whole-lung tissue and (**F**) relative density of CC3 to β-actin and (**G**) relative density of c-PARP to β-actin. Data represent mean ± SEM. ***P < 0.001, *P < 0.5 by unpaired *t* test. a IL-1β, anti–IL-1β.

To better investigate the functional effect of IL-1β on *K-ras*–mutant lung tumorigenesis, anti–IL-1β or isotype IgG control Ab was injected i.p. into a cohort of 6-week-old mice with *K-ras*^G12D^–mutant tumors (CC-LR mice) at a dose of 10 mg/kg twice a week for 8 weeks (Figure 1A). IL-1β blockade did not cause any noticeable change in mouse weight compared with the control group. Targeting IL-1β significantly decreased (by 40%) the number of lung-surface tumors (Figure 1B). Histopathologic analysis of lungs from treated mice revealed a smaller tumor or lung area (11.5% ± 1.5% vs. 17.2% ± 2%) (Supplemental Figure 1A; supplemental material available online with this article; https://doi.org/10.1172/jci.insight.157788DS1) and a lower percentage of adenoma or adenocarcinoma lesions (38.4% ± 5.8% vs. 54.9% ± 3.8%) (Supplemental Figure 1B). IL-1β blockade also suppressed the proliferation of tumor cells detected by lower Ki-67–positive staining (Figure 1C) and tumor angiogenesis presented by decreased expression of ERG (Figure 1D). IL-1β blockade also enhanced the apoptosis of cancer cells, characterized by increased expression of cleaved caspase-3 (CC3) and cleaved PARP (c-PARP) measured by Western blot (WB) (Figure 1, E–G).

### Immunopreventive blockade of IL-1β decreases protumor myeloid immune subsets in the TME.

**Figure 2:**
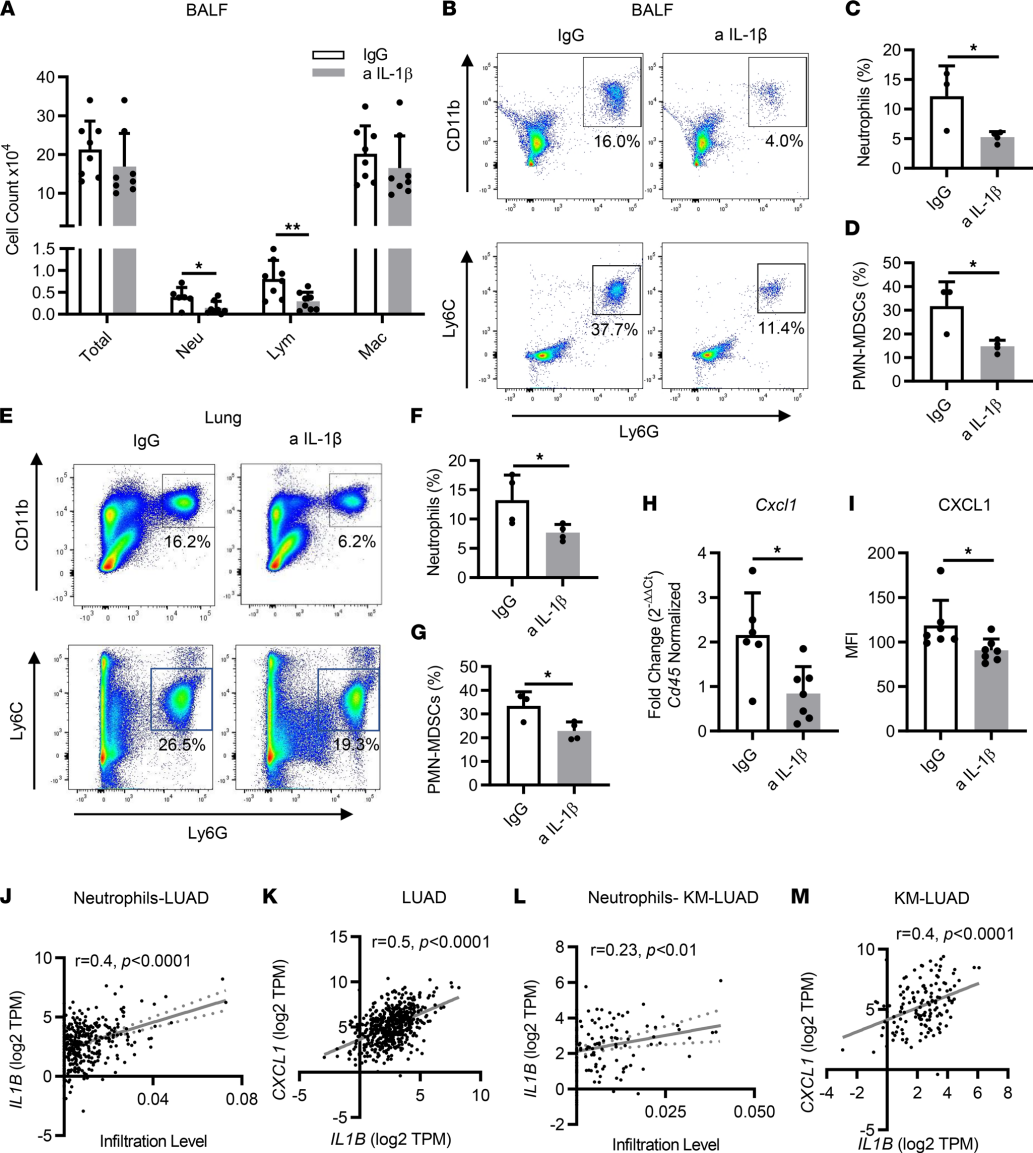
Immunopreventive blockade of IL-1β decreases protumor immune cells. (**A**) Total inflammatory cell and lineage-specific leukocyte numbers from BALFs of 14-week-old CC-LR mice treated with IgG or anti–IL-1β Ab (*n* = 8). (**B**–**G**) Representative flow cytometry analysis and quantification of neutrophils (CD11b^+^Ly6G^+^) and PMN-MDSCs (CD11b^+^Ly6C^lo^Ly6G^+^) in (**B**–**D**) BALFs and (**E**–**G**) whole lungs (*n* = 3–4). (**H**) Relative mRNA expression of *Cxcl1* in the whole lung normalized to *Cd45* expression (*n* = 6–7). (**I**) MFI of CXCL1 measured by multiplex ELISA in IgG or anti–IL-1β Ab–treated mice (*n* = 7). (**J**) Correlation between *IL1B* expression and infiltration of neutrophils in LUAD (*n* = 533). (**K**) Correlation between *IL1B* expression and *CXCL1* expression in LUAD (*n* = 535). (**L**) Correlation between *IL1B* expression and infiltration level of neutrophils in KM-LUAD (*n* = 138). (**M**) Correlation between *IL1B* expression and *CXCL1* expression in KM- LUAD (*n* = 139). Data represent mean ± SEM. **P < 0.01, *P < 0.5 by unpaired *t* test. a IL-1β, anti–IL-1β; Lym, lymphocytes; Mac, macrophages; Neu, neutrophils; TPM, transcript count per million.

To elucidate mechanisms through which IL-1β promotes tumor progression, we analyzed bronchoalveolar lavage fluids (BALFs) and whole lungs of CC-LR mice with or without IL-1β blockade to investigate the effects on tumor-infiltrating immune subsets within the TME. Wright-Giemsa staining of BALFs showed a significant decrease in neutrophils in anti–IL-1β–treated CC-LR mice, whereas macrophages and total WBC counts did not change (Figure 2A). Neutrophil reduction and no changes in macrophages were further confirmed by the flow cytometry (Figure 2, B and C, and Supplemental Figure 1C). Neutrophils represent an important component of the TME, where they can be immunosuppressive in the form of myeloid-derived suppressor cells (MDSCs) or directly tumor promoting (22, 23). We found a significant decrease in PMN-MDSCs (CD11b^+^Ly6C^lo^Ly6G^+^) in the BALFs of anti–IL-1β–treated CC-LR mice (Figure 2, B and D). Although the immune cell profile of BALF does not entirely represent the lung immune microenvironment, it reflects quantitative and phenotypic changes in the lung immune profile in response to a specific intervention. Similarly, flow cytometric analysis of the whole lung showed a significant decrease in neutrophils (Figure 2, E and F) and PMN-MDSCs (Figure 2, E and G) in anti–IL-1β–treated CC-LR mice. In addition, expression of the neutrophil chemoattractant CXCL1 was dramatically decreased in lungs of anti–IL-1β–treated CC-LR mice at the mRNA (Figure 2H) and protein (Figure 2I) levels, which may explain the observed reduction in neutrophils. Consistent with our observations in BALF, total macrophage counts were comparable between the 2 groups in the whole lung as well (Supplemental Figure 1D), along with no obvious macrophage polarization (Supplemental Figure 1, E and F).

Next, we corroborated these findings in human LUAD and KM-LUAD samples from The Cancer Genome Atlas (TCGA). Strong positive correlations were observed between *IL1B* expression and neutrophil infiltration (*r* = 0.4; *P* < 0.0001) or the expression of the neutrophil chemoattractant *CXCL1* (*r* = 0.5; *P* < 0.0001) in LUAD (Figure 2, J and K). Similarly, we found a markedly positive correlation between *IL1B* and *CXCL1* levels (*r* = 0.4; *P* < 0.0001) when we analyzed KM-LUADs (Figure 2M). We also noted a positive correlation between *IL1B* expression and neutrophil infiltration in KM-LUAD, albeit to a lesser extent, likely due to the smaller size of this LUAD subpopulation (*r* = 0.23; *P* < 0.01) (Figure 2L).

### Immunopreventive blockade of IL-1β modulates the TME toward an antitumor immune phenotype.

**Figure 3:**
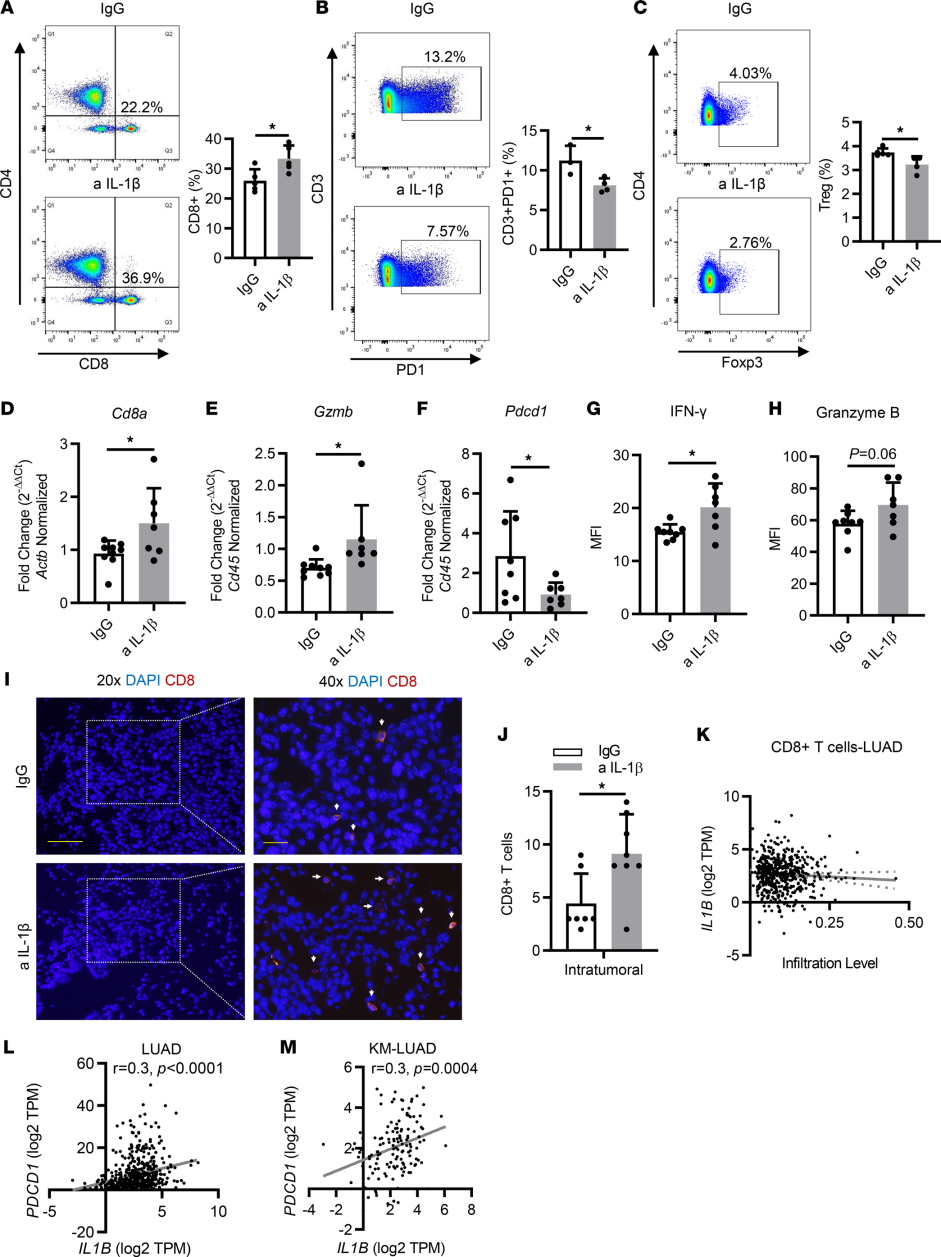
Immunopreventive blockade of IL-1β modulates the TME to an antitumor immune phenotype. (**A**) Representative flow cytometry analysis of CD8^+^ T cells and percentage of CD8^+^ T cells in the whole lung (*n* = 5). (**B**) Representative flow cytometry analysis of CD3^+^ programmed cell death 1–positive (PD-1^+^) T cells and percentage of CD3^+^PD-1^+^ T cells in the whole lung (*n* = 3–4). (**C**) Representative flow cytometry analysis of regulatory Tregs and percentage of Tregs in the whole lung (*n* = 4–5). (**D**–**F**) Relative mRNA expression of (**D**) *Cd8a*, (**E**) *Gzmb*, and (**F**) *Pdcd1* in the whole lung, normalized to *Cd45* or *Actb* expression (*n* = 7–9). (**G** and **H**) MFI of (**G**) IFN-γ and (**H**) granzyme B measured by multiplex ELISA in IgG or anti–IL-1β Ab–treated mice (*n* = 7–8). (**I**) Representative photomicrographs and (**J**) quantification of IF staining with anti-CD8a (red) and DAPI (blue) in tumor lesions (left top and bottom: original magnification, ×20, scale bar: 50 μm; right top and bottom: ×40, scale bar: 20 μm.) *n* = 7–8. (**K**) Correlation between *IL1B* expression and infiltration of CD8^+^ T cells in LUAD (*n* = 535). (**L**) Correlation between *IL1B* expression and *PDCD1* expression in LUAD (*n* = 535). (**M**) Correlation between *IL1B* expression and *PDCD1* expression in KM-LUAD (*n* = 139). Data represent mean ± SEM. *P < 0.5 by unpaired *t* test. a IL-1β, anti–IL-1β; TPM, transcript count per million.

Apart from myeloid immune subsets, we also looked at the frequency and phenotype of lymphocyte populations in the TME. Flow cytometric analysis of whole lungs showed that the total CD3^+^ T cell population was increased in the anti–IL-1β–treated mice (Supplemental Figure 1G). Among those T cells, we found a higher frequency of CD8^+^ T cells (Figure 3A), along with a reduction in phenotypically exhausted (PD-1^+^) T cells (Figure 3B). Conversely, no changes were observed in the total number of CD4^+^ T cells (Supplemental Figure 1H); however, subtype analysis of CD4^+^ populations revealed a significant decrease in the number of Tregs (CD4^+^Foxp3^+^) (Figure 3C), whereas no obvious changes were seen in Th1, Th2, and Th17 populations (data not shown).

Of note, to better profile the gradient between cytotoxic T cells and myeloid immunosuppressive cells in the lung microenvironment, we also determined the ratios of IFN-γ–expressing CD8^+^ T cells (or cytotoxic T lymphocytes [CTLs]) to neutrophils or PMN-MDSCs among all the CD45^+^ populations. A significantly elevated value of CTLs/neutrophils ratio was observed in anti–IL-1β–treated CC-LR mice (Supplemental Figure 1I), and the same applied to the ratio of CTLs to PMN-MDSCs (data not shown).

Quantitative real-time PCR (qRT-PCR) analysis of RNA extracted from the whole lung further confirmed an increase in *Cd8a* expression (Figure 3D), whereas no change was seen in *Cd4* expression (Supplemental Figure 1J), which is consistent with our flow cytometry results. Moreover, higher *Cd8a* expression was accompanied by the elevated expression of *Gzmb* and decreased expression of *Pdcd1*(Figure 3, E and F), which suggests higher infiltration of cytotoxic T cells. Furthermore, multiplex ELISA of whole-lung protein extract showed a significantly increased expression of IFN-γ (Figure 3G) and granzyme B (Figure 3H), which further validated enhanced antitumor cytotoxic activity after IL-1β blockade.

Subsequently, we assessed T cell spatial distribution by immunofluorescence (IF) staining. We found profuse infiltration of CD8^+^ T cells in tumors in IL-1β blockade mice, whereas these cells were scarce in tumors of control mice (Figure 3, I and J). In addition, representative multiplex IHC staining revealed a greater proportion of intratumoral T cells expressing PD-1 in the control group compared with anti–IL-1β–treated CC-LR mice (Supplemental Figure 2A).

After analysis of the TCGA cohort, we found a trend, albeit not statistically significant, for a negative correlation between CD8^+^ T cell infiltration and *IL1B* expression in patients with LUAD (Figure 3K). Of note, we found that *PDCD1* (coding for PD-1) expression levels were significantly and positively correlated with *IL1B* levels in LUAD (*r* = 0.3; *P* < 0.0001) as well as in KM-LUAD (*r* = 0.3; *P* = 0.0004) (Figure 3, L and M).

### Early therapeutic effects of IL-1β blockade on KM-LUAD.

**Figure 4:**
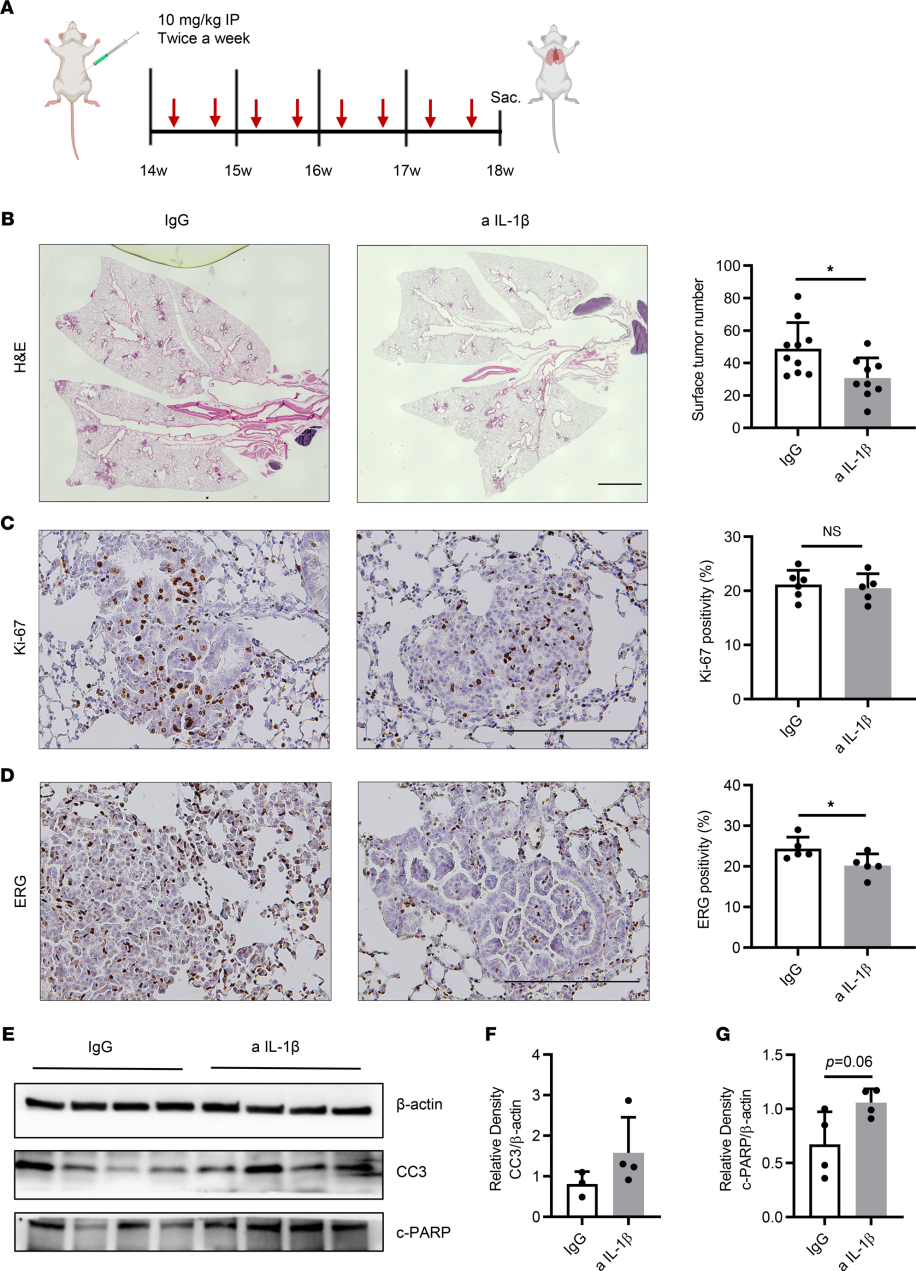
Immunotherapeutic targeting of IL-1β decreases tumor burden, decreases tumor angiogenesis, and increases tumor cell apoptosis. (**A**) Schematic illustration of the experimental plan and procedure. CC-LR mice received IgG or anti–IL-1β Ab at the age of 14 weeks and were sacrificed at 18 weeks of age. (**B**) Lung surface tumor number (*n* = 9–10) and photomicrographs of the H&E–stained lung sections in 18-week-old CC-LR mice treated with IgG or anti–IL-1β Ab. Original magnification, ×4. Scale bar: 2 mm. (**C**) Representative photomicrographs of Ki-67–stained sections (original magnification, ×20; scale bar: 200 μm) and quantification of Ki-67 staining presented as the percentage of Ki-67–positive cells (*n* = 5–6). (**D**) Representative photomicrographs of ERG-stained sections (original magnification, ×20; scale bar: 200 μm) and quantification of ERG staining presented as the percentage of ERG-positive cells (*n* = 5–6). (**E**–**G**) WB analysis of CC3, c-PARP, and β-actin protein levels in whole-lung tissue (**E**) and relative density of CC3 to β-actin (**F**) and c-PARP to β-actin (**G**). Data represent mean ± SEM. *P < 0.5 by unpaired *t* test. a IL-1β, anti–IL-1β.

Based on our findings that IL-1β blockade can strengthen antitumor cytotoxic activity in the TME, we also sought to determine the early therapeutic effects of IL-1β blockade in *K-ras*–mutant lung cancer. We blocked IL-1β by i.p. delivery of anti–IL-1β Ab into a cohort of 14-week-old mice at a dose of 10 mg/kg twice a week for a total of 4 weeks (Figure 4A). At 18 weeks of age, we found that lung-surface tumor count was reduced by 37.1% compared with that in control counterparts (Figure 4B). Histopathologic analysis of lungs from anti–IL-1β–treated mice revealed that the percentages of lung area occupied by tumor and adenoma and adenocarcinoma lesions were reduced by 47.5% and 53%, respectively (Supplemental Figure 3, A and B). In addition, although the level of the proliferation marker Ki-67 was similar between the 2 groups (Figure 4C), there was decreased tumor angiogenesis in treated mice, as indicated by lower ERG-positive staining (Figure 4D). WB analysis showed a slight increase in expression of CC3 and a significant increase in c-PARP expression (Figure 4, E–G), indicating that the direct impact of 4 weeks of IL-1β blockade on apoptosis was less prominent in established tumors during the therapeutic intervention compared with the preventive setting (Figure 1, E–G).

### Immunotherapeutic blockade of IL-1β increased cytotoxic T cell infiltration.

**Figure 5:**
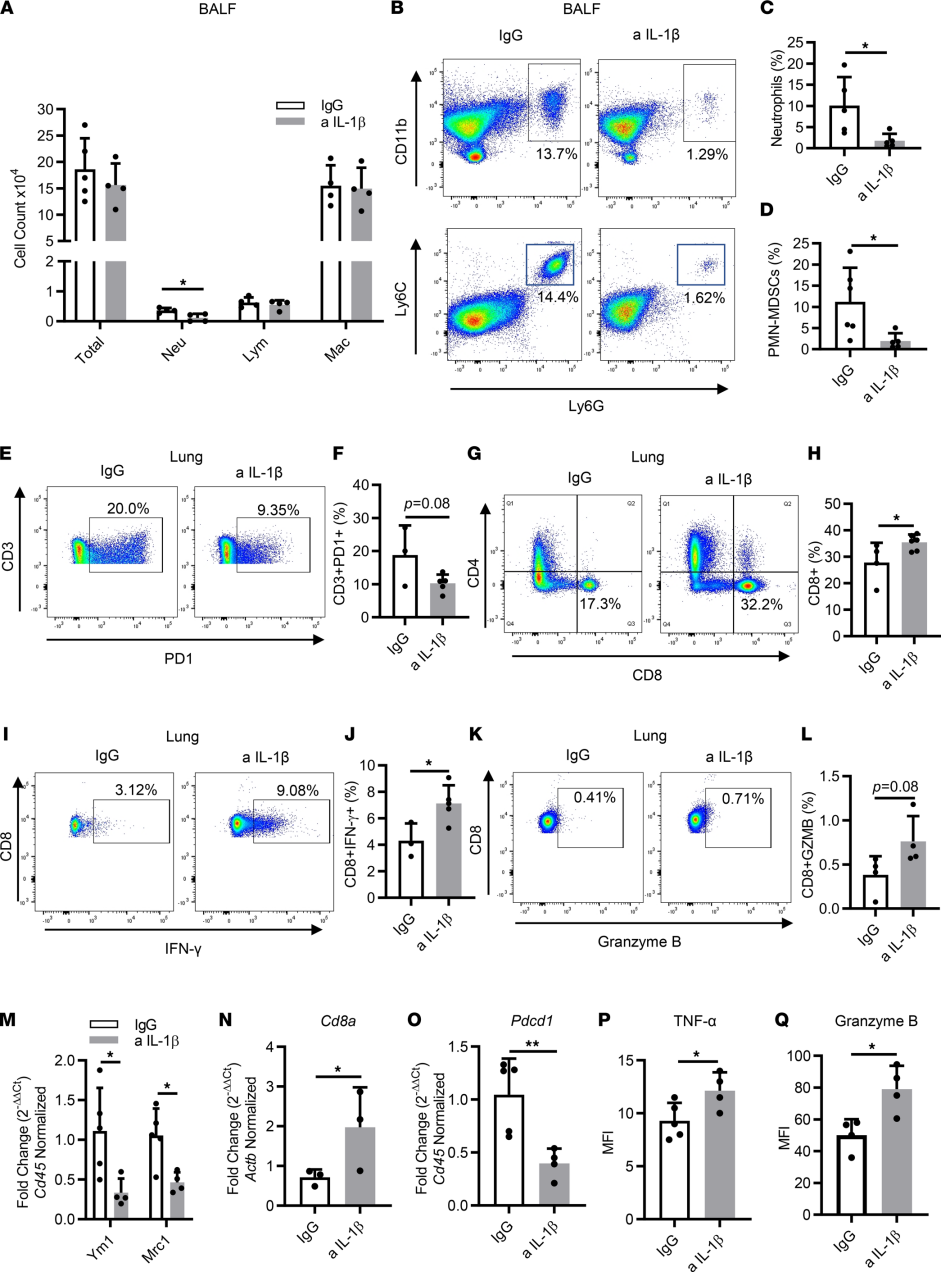
Immunotherapeutic blockade of IL-1β increases activated T cells’ infiltration. (**A**) Total inflammatory cell and lineage-specific leukocyte numbers from BALFs of 18-week-old CC-LR mice treated with IgG or anti–IL-1β Ab (*n* = 4–5). (**B**) Representative flow cytometry analysis of neutrophils (CD11b^+^Ly6G^+^) and PMN-MDSCs (CD11b^+^Ly6C^lo^Ly6G^+^) in BALFs. (**C** and **D**) Quantification percentage of (**C**) neutrophils and (**D**) PMN-MDSCs in BALFs (*n* = 5–6). (**E**–**L**) Representative flow cytometry analysis and quantification percentage of CD3^+^PD-1^+^ T cells (**E** and **F**), CD8^+^ T cells (**G** and **H**), IFN-γ–expressing CD8^+^ T cells (**I** and **J**), and granzyme B–expressing CD8^+^ T cells (**K** and **L**) in whole lungs of 18-week-old CC-LR mice treated with IgG or anti–IL-1β Ab (*n* = 3–6). (**M**–**O**) Relative mRNA expression of *Ym1* and *Mrc1* (**M**), *Cd8a* (**N**), and *Pdcd1* (**O**) in the whole lung, normalized to *Cd45* or *Actb* expression (*n* = 3–5). (**P** and **Q**) MFI of TNF-α (**P**) or granzyme B (**Q**) measured by multiplex ELISA in IgG or anti–IL-1β–treated mice (*n* = 4–5). Data represent mean ± SEM. **P < 0.01, *P < 0.5 by unpaired *t* test. a IL-1β, anti–IL-1β; Lym, lymphocytes; Mac, macrophages; Neu, neutrophils.

Next, we sought to investigate how IL-1β blockade affects the TME in a therapeutic setting. We first looked at BALFs and found a significant decrease in neutrophils, whereas the numbers of lymphocytes and macrophages remained similar to those of the control group (Figure 5A). Flow cytometry confirmed the reduction of neutrophils (Figure 5, B and C) and no change in the proportion of macrophages in the BALFs (Supplemental Figure 3C). In addition, a sharp decrease in the percentage of PMN-MDSCs was observed (Figure 5, B and D). Flow cytometric analysis of the whole lung also revealed macrophage populations were not altered between the 2 groups (Supplemental Figure 3D); however, we found a significant reduction in the percentage of M2 macrophages, indicating that IL-1β inhibition drives antitumor M1 macrophage polarization (Supplemental Figure 3E).

To better understand the lymphocyte profile, we subsequently performed flow cytometry of the whole lung with lymphocytic parameters. Among all the CD3^+^ T cells, PD-1^+^ populations were decreased after IL-1β blockade (Figure 5, E and F). Representative multiplex IHC staining revealed a higher number of double-positive CD3^+^PD-1^+^ T cells residing in the tumor in the control group compared with the anti–IL-1β–treated CC-LR mice (Supplemental Figure 4A). Meanwhile, there was a significant increase in CD8^+^ T cells in anti–IL-1β–treated mice, whereas no changes were observed in CD4^+^ populations (Figure 5, G and H, and Supplemental Figure 4B). As confirmed by IF staining, ample CD8^+^ T cells were observed in the tumors of mice that received anti–IL-1β treatment, whereas fewer were present in the control tumors (Supplemental Figure 4, C and D). The functional parameters related to tumor-infiltrating CD8^+^ T cells were assessed using intracellular staining of IFN-γ and granzyme B. We observed significantly increased intracellular expression of IFN-γ (Figure 5, I and J) and slight elevation of granzyme B expression (Figure 5, K and L) in whole lungs of mice with IL-1β inhibition, compared with control counterparts. In addition, the ratios of CTLs to neutrophils (Supplemental Figure 4E) and CTLs to PMN-MDSCs (data not shown) were dramatically increased in IL-1β–blockade mice. Collectively, these results elucidated that immunotherapeutic blockade of IL-1β increases cytotoxic T cell infiltration in lung tumors.

We further validated our observations of alterations in infiltrating leukocytes by qRT-PCR. We first looked at the expression level of M1 and M2 macrophage–associated genes in whole lungs. There was a robust reduction in expression of *Ym1* and *Mrc1* (Figure 5M), which aligned well with the M2 subset reduction observed by flow cytometry. In addition, a significant increase in *Cd8a* expression was found (Figure 5N), which was accompanied by a significant decrease in *Pdcd1* expression (Figure 5O), whereas *Cd4* expression remained the same (Supplemental Figure 4F), which is in line with our flow cytometry results. Moreover, multiplex ELISA analysis in the whole lungs of CC-LR mice after IL-1β inhibition revealed an increase in TNF-α and granzyme B expressions (Figure 5, P and Q). Taken together, these findings suggested that IL-1β blockade re-educates the TME to an antitumor ambiance.

### IL-1β blockade effectively inhibits the NF-κB and STAT3 pathways in KM-LUAD.

**Figure 6:**
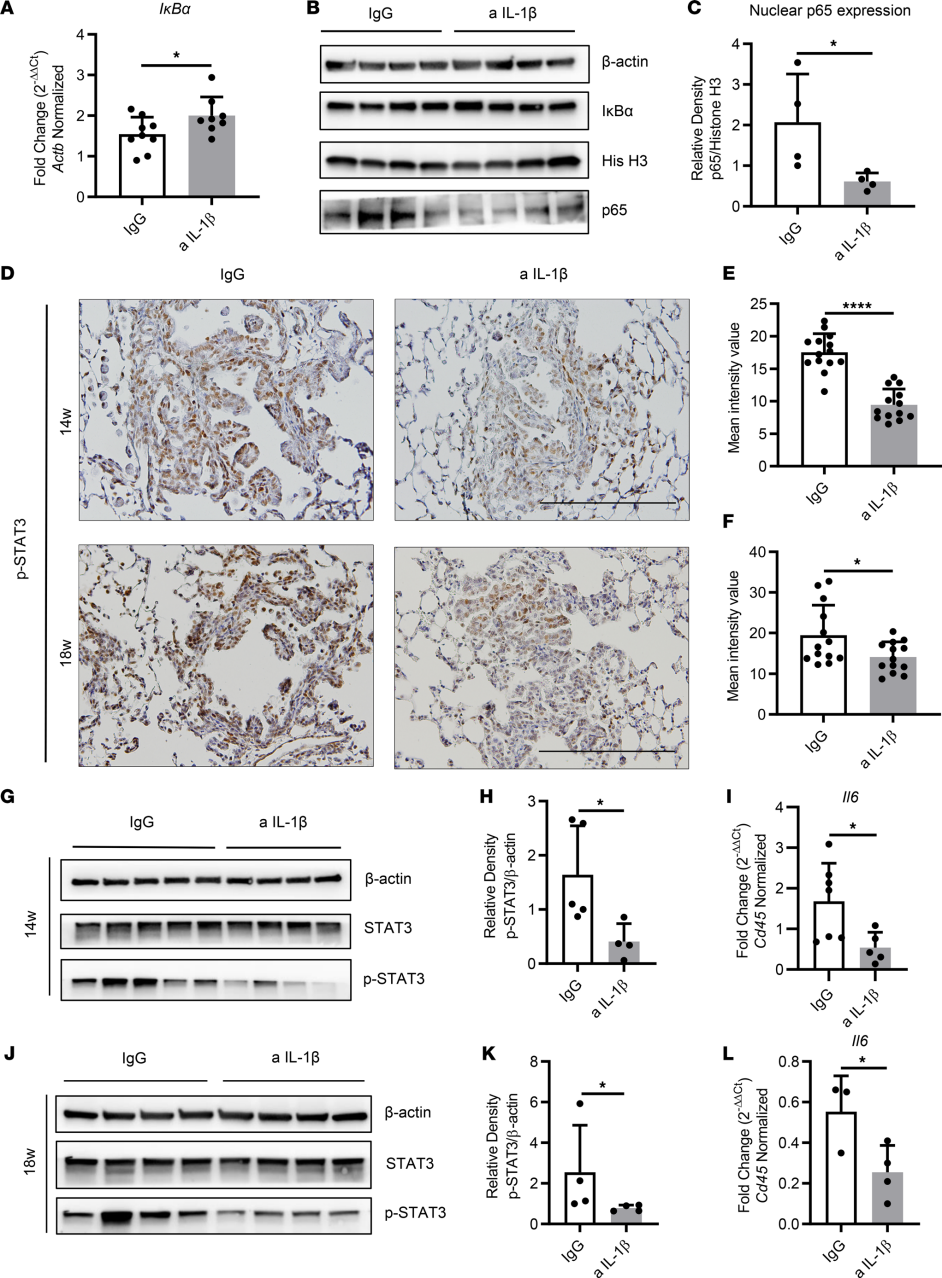
IL-1β blockade effectively inhibits NF-κB and/or STAT3 pathway in KM-LUAD. (**A**) Relative mRNA expression of *IκBα* in the whole lungs of 14-week-old CC-LR mice, normalized to *Actb* expression (*n* = 8–9). (**B**) WB analysis of IκBα and β-actin protein levels in whole lung tissue (note: the β-actin band was shared with Figure 1E because they were blotted with the same samples) and p65, histone H3 (His H3) protein levels in whole-lung tissue nuclear extracts of 14-week-old CC-LR mice. (**C**) Relative density of p65 to histone H3 in nuclear extracts from the whole lungs. (**D**) Representative photomicrographs of phosphorylated STAT3–stained sections (original magnification, ×20; scale bar: 200 μm) in IgG or anti–IL-1β Ab–treated CC-LR mice at the age of 14 or 18 weeks. (**E** and **F**) Quantification of phosphorylated STAT3 staining presented as mean intensity value (*n* = 5–8). (**G**–**K**) WB analysis of phosphorylated STAT3, total STAT3, and β-actin protein levels in whole-lung tissue and relative density of phosphorylated STAT3 to β-actin in IgG or anti–IL-1β Ab–treated CC-LR mice at the age of 14 (**G** and **H**) and 18 weeks (**J** and **K**). (**I** and **L**) Relative mRNA expression of *Il6* in the whole lungs of 14 (**I**) and 18-week-old (**L**) CC-LR mice, normalized to *Cd45* (*n* = 3–7). Data represent mean ± SEM. ****P < 0.0001, *P < 0.5 by unpaired *t* test.

Previous studies revealed that IL-1β is a potent activator of the NF-κB pathway (13, 14), and our group also demonstrated essential roles for NF-κB–mediated production of cytokines in the promotion of *K-ras*–mutant lung cancer in CC-LR mice (10, 24, 25). Here, we found that IL-1β blockade for 8 weeks effectively inhibited the NF-κB pathway, which was evidenced by an increased level of the NF-κB inhibitor *IκBα* (Figure 6A), the weaker binding activity of the p65 subunit (Supplemental Figure 5A), and a significantly decreased expression of p65 in nuclear extracts (Figure 6, B and C). Surprisingly, 4 weeks of treatment with anti–IL-1β did not significantly inhibit the NF-κB pathway, as evidenced by comparable mRNA and protein expression of IκBα (Supplemental Figure 5, B and C) and nuclear p65 expression (Supplemental Figure 5C).

STAT3 is another important inflammation pathway linked to *K-ras* mutation, and NF-κB–regulated cytokines such as IL-6 mostly activate the STAT3 pathway (26, 27). In line with our NF-κB findings, IHC revealed a largely decreased expression of phosphorylated STAT3 (p-STAT3) in the tumor lesions of anti–IL-1β–treated mice at the age of 14 or 18 weeks (Figure 6, D–F). Lower p-STAT3 expression was further confirmed by WB analysis of whole-lung protein, whereas total STAT3 expression remained similar between the 2 groups (Figure 6, G, H, J, and K), suggesting that the STAT3 pathway was effectively restrained by IL-1β blockade following both 4 and 8 weeks of treatments. Moreover, qRT-PCR analysis of whole-lung RNA from 14- and 18-week-old mice revealed a significant decrease in expression of *Il6* (Figure 6, I and L), a known product of the NF-κB pathway and a potent activator of the STAT3 pathway, which could explain the observed reduction in STAT3 pathway activation.

## Discussion

KM-LUAD is strongly associated with inflammation characterized by infiltration of various immune cells and enrichment of versatile cytokines (28). IL-1β is a proinflammatory cytokine and has emerged as a therapeutic target for several inflammatory diseases, such as rheumatoid arthritis (29, 30); however, its role in the development of lung cancer, especially KM-LUAD, is not fully understood. Here, we explored the therapeutic, and particularly the preventive, effects of anti–IL-1β in a mouse model with *K-ras*–mutant lung tumors (CC-LR mice). We found that IL-1β inhibition decreased tumor burden, tumor cell proliferation, and tumor angiogenesis, while increasing tumor cell apoptosis from both preventive and early therapeutic perspectives. IL-1β blockade led to increased cytotoxic CD8^+^ T cell infiltration and a curbed protumor immunosuppressive response. This treatment also effectively inhibited the activation of the NF-κB and STAT3 pathways.

The CANTOS phase III randomized clinical trial was designed to evaluate the role of IL-1β inhibition in preventing the recurrence of cardiovascular events in patients with atherosclerosis (21); unexpectedly, treatment with the anti–IL-1β mAb canakinumab resulted in a dose-dependent reduction in lung cancer incidence and mortality. This study, although primarily designed as a cardiovascular outcomes trial, provided evidence that IL-1β inhibition may potentially benefit individuals at high risk for lung cancer. It is important to note that CANTOS trial participants harbored persistent proinflammatory responses, defined by the presence of high-sensitivity C-reactive protein with concentrations of 2 mg/L or greater, which were reduced by the given treatment, and greater than 70% of patients were past or current smokers, indicating anti–IL-1β treatment likely reduced the inflammation that progresses to carcinogenesis. These patient characteristics demonstrate the applicability of IL-1β inhibition as a potential strategy for prevention or treatment of KM-LUAD in which inflammation is highly involved in tumor development and progression. Moreover, based on findings from an ongoing CANOPY-1 (Study of Efficacy and Safety of Pembrolizumab Plus Platinum-based Doublet Chemotherapy With or Without Canakinumab in Previously Untreated Locally Advanced or Metastatic Non-squamous and Squamous NSCLC Subjects) trial (31), researchers evaluating canakinumab as a first-line treatment for locally advanced or metastatic NSCLC in combination with pembrolizumab- and platinum-based doublet chemotherapy reported that clinically meaningful improvements in both progression-free survival (PFS) and overall survival (OS) were observed among a subset of patients with inflammatory evidence, although the trial did not meet its primary endpoints of PFS and OS in all patients. This observation further supports the importance of IL-1β blockade as a preventive approach primarily and/or for adequately screening a subgroup of patients with lung cancer who may potentially benefit from anti-IL-1β treatment.

IL-1β is abundant at tumor sites, and malignant and normal epithelial cells, as well as fibroblasts and immune cells, are all considered potential sources of IL-1β in different tumor models (32–34). Myeloid cells were reported as the primary source of IL-1β in human NSCLC samples from single-cell RNA-Seq data sets and, accordingly, were demonstrated in mouse models (35, 36). As a pleiotropic cytokine, IL-1β has been reported to promote tumors by influencing different subsets of immune cells; a well-accepted mechanism is the recruitment of protumor myeloid cells (37). In previous studies, researchers reported that IL-1β drives neutrophilic influx and modulates lung inflammation, and an apparent decrease of neutrophils was observed in IL-1β^–/–^ mice with chemical carcinogen–induced tumors (38, 39). Neutrophils are involved in tumor initiation or progression directly through remodeling extracellular matrix by production of factors such as neutrophil elastase (40, 41) or influencing immune surveillance (42). We and others have previously demonstrated that neutrophils depletion from the lung TME suppresses lung tumorigenesis in several mouse models and particularly in models of KM-LUAD (11, 40, 43–47). Neutrophils can also indirectly affect the infiltration and function of cytotoxic T cells via secreting neutrophil elastase (41) and undergoing NETosis to form neutrophil extracellular traps (48). Kargl et al. (49) found a strong negative correlation between neutrophils and CD8^+^/CD4^+^ lymphocytes in NSCLC samples. In this study, we found IL-1β neutralization dramatically decreased the number of neutrophils both in BALFs and whole lungs. This reduction in neutrophils partly explains the increased infiltration of cytotoxic T cells in tumors. In addition to neutrophils, MDSCs, which comprised PMN-MDSC and monocytic MDSC subsets, represent a diverse population of immature myeloid lineage cells that contributes to negative regulation of the immune response during cancer (23). IL-1β–induced suppression of antitumor immune responses through the recruitment of MDSCs was demonstrated in various cancer models (15, 17, 32, 50, 51). MDSCs can promote tumor progression by suppressing T cell function, recruiting Tregs, and polarizing macrophages toward an M2 phenotype (23, 52, 53). In our study, flow cytometry revealed a significant decrease in PMN-MDSCs (CD11b^+^Ly6C^lo^Ly6G^+^) and M2-type macrophages after IL-1β blockade, which could explain the reduction of exhausted T cells in lung tumors.

In addition, we validated the correlation between *Il1B* expression and TME profile in tumor tissue derived from patients with LUAD. Both neutrophil infiltration and neutrophil chemokine *CXCL1* expression had an overall strong, positive concordance with *Il1B* expression in LUAD, which mirrors the results we obtained from mice, and further indicates that the enrichment level of neutrophils may help better define the patients who would benefit from anti–IL-1β therapy. Immune checkpoint inhibitors (ICIs) have revolutionized treatment of multiple types of cancer, including NSCLC (54). KM-LUAD is strongly associated with inflammation, rendering immunotherapy a desirable strategy for tackling this disease; however, only a limited number of patients benefit from ICIs (55). It was recently reported that poor tumor infiltration by CD8^+^ T cells and increased neutrophil to lymphocyte ratio were negatively correlated with PFS as well as OS of patients treated with ICIs (56, 57). High preoperative neutrophil to lymphocyte ratio was significantly related to *K-ras* mutation and associated with shorter OS in patients with resected NSCLC (58). In our study, a significantly increased ratio of CTLs to neutrophils was observed in the anti–IL-1β Ab–treated mice, and IL-1β blockade on its own increased infiltration of CTLs, evidenced by increased expression of IFN-γ and granzyme B, along with markedly decreased expression of *PD-1* (*Pdcd1*). Of note, a positive correlation between serum IL-1β levels and tumoral *PD-1* (*PDCD1*) expression was previously demonstrated in patients with LUAD (58). These results suggest IL-1β inhibition may be a potential alternative therapeutic modality in both early and later stages of KM-LUAD that does not respond to ICIs. Given that IL-1β blockade increased the infiltration of CD8^+^ T cells with robust cytotoxic activity, perhaps combinatorial approaches with conventional chemotherapy, radiotherapy, or targeted therapy (e.g., MEK inhibitor), or even with surgical resection in adjuvant or neoadjuvant settings, could enhance its antitumor effects.

Taken together, we demonstrated that IL-1β blockade effectively suppresses *K-ras*–mutant lung tumorigenesis by shifting the immunosuppressive TME to an antitumor phenotype, possibly via modulating the NF-κB and STAT3 pathways. Our results support IL-1β as a potential target with preventive and therapeutic benefits for patients with KM*-*LUAD.

## Methods

### Mice.

CCSP^Cre^/LSL-*K-ras*^G12D^ (CC-LR) mice were generated as previously described (60). Briefly, 6-week-old mice harboring the LSL-*K-ras*^G12D^ allele were crossed with mice carrying Cre recombinase inserted into the club cell secretory protein (CCSP) locus (CCSP^Cre^ mice; both on a C57BL/6 background), which led to lung epithelial–specific expression of mutant *K-ras*. All mice were housed under specific pathogen–free conditions and handled in accordance with the guidelines of the IACUC of MD Anderson Cancer Center. Mice were monitored daily for evidence of disease or death.

### In vivo IL-1β blockade.

For immunoprevention, 6-week-old CC-LR mice were injected i.p. with 10 mg/kg anti–IL-1β mAb (clone 01BSUR, provided by Novartis) or IgG1 isotype control Ab (clone 112687, provided by Novartis) twice a week from 6 to 14 weeks of age (Figure 1A). Therapeutic effects were probed by i.p. injection of the same anti–IL-1β mAb or IgG1 isotype control Ab with the same regimen, but from 14 to 18 weeks of age, as schematically depicted in Figure 4A.

### Assessment of lung tumor burden and inflammation.

Mice were anesthetized by i.p. injection of avertin (0.8 mL, 0.25 mg/mL; Sigma), and their tracheas were cannulated and sutured into place at the age of 14 or 18 weeks. Lung surface tumor number was counted, if the tumors were visible. In half of the mice, lungs were perfused through the right ventricle with PBS, inflated with 10% buffered formalin (Sigma) for 10 minutes, and collected for histological studies. H&E–stained sections were prepared from 5 to 8 animals per group, and 5 randomly selected microscopic fields of the lungs were photographed. The percentage of the lung field occupied by tumors was measured by overlaying these images on a dotted grid, as we previously described (25). In addition, each lesion was assessed as either atypical adenomatous hyperplasia or adenoma/adenocarcinoma, and the proportion was calculated. From the other half of the mice, BALFs were collected by sequentially instilling and collecting 2 aliquots of 1 mL of PBS through a tracheostomy cannula. Subsequently, the lungs were perfused with PBS, snap frozen, and stored for RNA or protein analysis. Total WBC count in BALFs was determined using a hematocytometer, and differential cell populations were identified by cytocentrifugation of BALFs and subsequent Wright-Giemsa (Sigma) staining.

### Isolation of lung inflammatory cells by flow cytometry.

Mice were treated as described above until the ages of 14 or 18 weeks. Lungs were perfused with PBS, collected, cut into a homogenous paste, and then digested with 1 mg/mL collagenase IV (Gibco) in RPMI (GenDEPOT) for 30 minutes at 37°C. Single-cell suspensions were prepared by mechanical dissociation of lung tissue through a 70 μm nylon mesh (Falcon). RBC lysis was performed for 1–3 minutes, if needed. Lung cells were suspended in FACS buffer (composed of 1x PBS [Sigma], and 4mL of 0.5M EDTA [MilliporeSigma] at a final concentration of 2mM, plus 10mL FBS [GenDEPOT] at a final concentration of 1%) for surface staining. Cell-surface staining was conducted by incubating cells with Abs for 30 minutes on ice in the presence of anti-CD16/CD32 (clone 2.4G2, Tonbo) to block FcγR binding. To assess T cell cytokine production, 3 × 10^6^ cells were plated in 6-well plates and were stimulated with 50 ng/mL phorbol 12-myristate 13-acetate (Sigma) and 500 ng/mL ionomycin (Sigma), and vesicular trafficking was blocked with 1 μL/mL GolgiStop (BD Biosciences) and 1 μL/mL GolgiPlug (BD Biosciences) for 4 hours at 37°C. Cells were stained for surface markers and then permeabilized using a transcription factor–staining kit (Invitrogen) to detect intracellular cytokines, FoxP3, and granzyme B. All Abs used are listed in Supplemental Table 1. All data were acquired using an LSRFortessa X-20 (BD) and analyzed with FlowJo software, version 10 (Tree Star). The gating strategies are shown in Supplemental Figure 6, A and B.

### IHC/IF staining.

H&E and IHC staining for proliferation marker Ki-67 (1:200; catalog ab16667, Abcam), angiogenesis marker ERG (1:200; catalog ab92513, Abcam), and p-STAT3 (Tyr705) (1:200; catalog 9145S, Cell Signaling Technology) were performed as previously described (10). CD8a (1:100; catalog 98941S, Cell Signaling Technology) was stained according to a standard IF protocol. For the quantification of Ki-67 and ERG, the ratios of positive to total tumor cells per ×20 field were measured using ImageJ (NIH) and expressed as percentages. p-STAT3 was quantified as previously described (61).

### Fluorescent multiplex IHC consecutive staining on a single slide.

The Opal 7-Color Manual IHC Kit (AKOYA Biosciences, NEL811001KT) was used for multiplex IHC consecutive staining on a single slide. A series of sequential cycles of blocking, epitope retrieval, primary and secondary Ab incubation, and opal fluorophore staining were performed on FFPE tissue samples. We visualized 3-color Opal slides through Mantra or Vectra Quantitative Pathology Imaging Systems. The systems use multispectral imaging for quantitative unmixing of fluorophores and tissue autofluorescence. The primary Abs used for multiplex IHC were anti-CD3ε (1:100; catalog 78588, Cell Signaling Technology) and anti–PD-1 (1:150; catalog 84651, Cell Signaling Technology).

### qRT-PCR analysis.

Total RNA from the whole lung was isolated using the E.Z.N.A total RNA kit (Omega) according to the manufacturer’s protocol. qRT-PCR was conducted using the qScript cDNA SuperMix (Quanta Biosciences). qRT-PCR was performed using SYBR Green FastMix (Quanta Biosciences) on the CFX96 Touch Real-Time PCR Detection System (Bio-Rad). Fold changes between test groups and controls were calculated using the 2^–ΔΔCt^ method. Gene-specific primers are listed in Supplemental Table 2.

### WB analysis.

Lung tissue was homogenized in RIPA buffer (Sigma) and a protease inhibitor (Thermo Fisher Scientific) mixture. After incubation for 30 minutes on ice, samples were centrifuged at 18,620 x g for 30 minutes at 4°C, and supernatants were collected as total protein. Nuclear proteins were extracted using NE-PER Nuclear Protein Extraction Kit (Pierce) according to the manufacturer’s instructions. Protein concentrations were determined using the bicinchoninic acid assay (Thermo Fisher Scientific). WB was performed as previously described (24). The primary Abs used were against IκBα (1:1000; catalog ab32518, Abcam), p65 (1:400; catalog 1546-1, Epitomics), CC3 (1:1000; catalog 9664S, Cell Signaling Technology), c-PARP (1:1000; catalog 9544S, Cell Signaling Technology), STAT3 (1:1000; catalog 4904S, Cell Signaling Technology), p-STAT3 (Tyr705; 1:1000; catalog 9145S, Cell Signaling Technology), histone H3 (1:1000; catalog 4499S, Cell Signaling Technology), and β-actin (1:1000; catalog 4970S, Cell Signaling Technology).

### Cytokine/chemokine ELISA measurement.

Cell Lysis Buffer 2 (R&D Systems) was used to extract total protein from whole-lung tissue, and 110 μg of protein per sample was subsequently analyzed. Quantification of cytokine concentrations, including CXCL1, TNF-α, IFN-γ, and granzyme B in lung lysates, was performed using the Mouse Magnetic Luminex Assay (catalog LXSAMSM; R&D Systems) according to the manufacturer’s instructions. MFI was measured using Luminex MAGPIX (R&D Systems).

### Measurement of NF-κB activity in lung tissues.

The NF-κB (p65) Transcription Factor Assay Kit (Cayman Chemical Company) was used to measure NF-κB (p65) binding activity according to the manufacturer’s instructions, with 20 μg of nuclear extract in duplicates. OD was measured with a microplate reader set to 450 nm and represented as the activity of p65 binding ability.

### TCGA data set analysis.

Somatic mutation and gene expression data of patients with LUAD were obtained from TCGA (https://portal.gdc.cancer.gov/). Immune profiles were evaluated by the CIBERSORT algorithm in the R statistical language and environment (version 3.5.1; R Project for Statistical Computing) (62). Linear regression analysis was performed to assess the correlation between the expression of 2 genes or infiltration level of immune cells and *IL1B* expression.

### Statistics.

Data are presented as mean ± SEM. The statistical significance between the 2 groups was calculated by a 2-tailed *t* test, with *P* < 0.05 defined as statistically significant. All statistical analyses were performed using SPSS, version 19.0 (IBM Corp.).

### Study approval.

All mice were handled in accordance with the guidelines of the IACUC of MD Anderson Cancer Center (approved protocol no. 00000993-RN02).

## Author contributions

SJM conceptualized the study and contributed to the methodology and supervision of the study, and reviewed and edited the manuscript. HK and SJM contributed to funding acquisition. BY, MJC, WVV, SP, QP, YS, MRC, MZ, SY, RLB, JVH, JZ, EJO, and HK contributed to project administration. BY, MJC, SP, SHC, and SSW curated the data and conducted the formal data analysis. BY and MJC contributed to study methodology, used the required software to analyze and conduct visualization techniques, and wrote the original draft of the manuscript. MJC, WVV, SP, MZ, HK, and SJM contributed to data validation and reviewed and edited the manuscript.

## Supplementary Material

Supplemental data

## Version 1 04/26/2022

In-Press Preview

## Version 2 06/08/2022

Electronic publication
