# Supplementary material for: Targeting IL-1β as an immunopreventive and therapeutic modality for K-ras–mutant lung cancer
Source: JCI Insight. 2022 Jun 8;7(11):e157788. doi: 10.1172/jci.insight.157788 (PMC9220853; doi:10.1172/jci.insight.157788)
Supplement: Supplemental data [file jciinsight-7-157788-s071.pdf]

Targeting IL-1β as an Immune Preventive and Therapeutic Modality for *K-ras* Mutant Lung Cancer

Bo Yuan, Michael J. Clowers, Walter V. Velasco, Stephen Peng, Qian Peng, Yewen Shi, Marco Ramos-Castaneda, Melody Zarghooni, Shuanying Yang, Rachel L. Babcock, Seon Hee Chang, John V. Heymach, Jianjun Zhang, Edwin J. Ostrin, Stephanie S. Watowich, Humam Kadara, Seyed Javad Moghaddam

Online Data Supplement

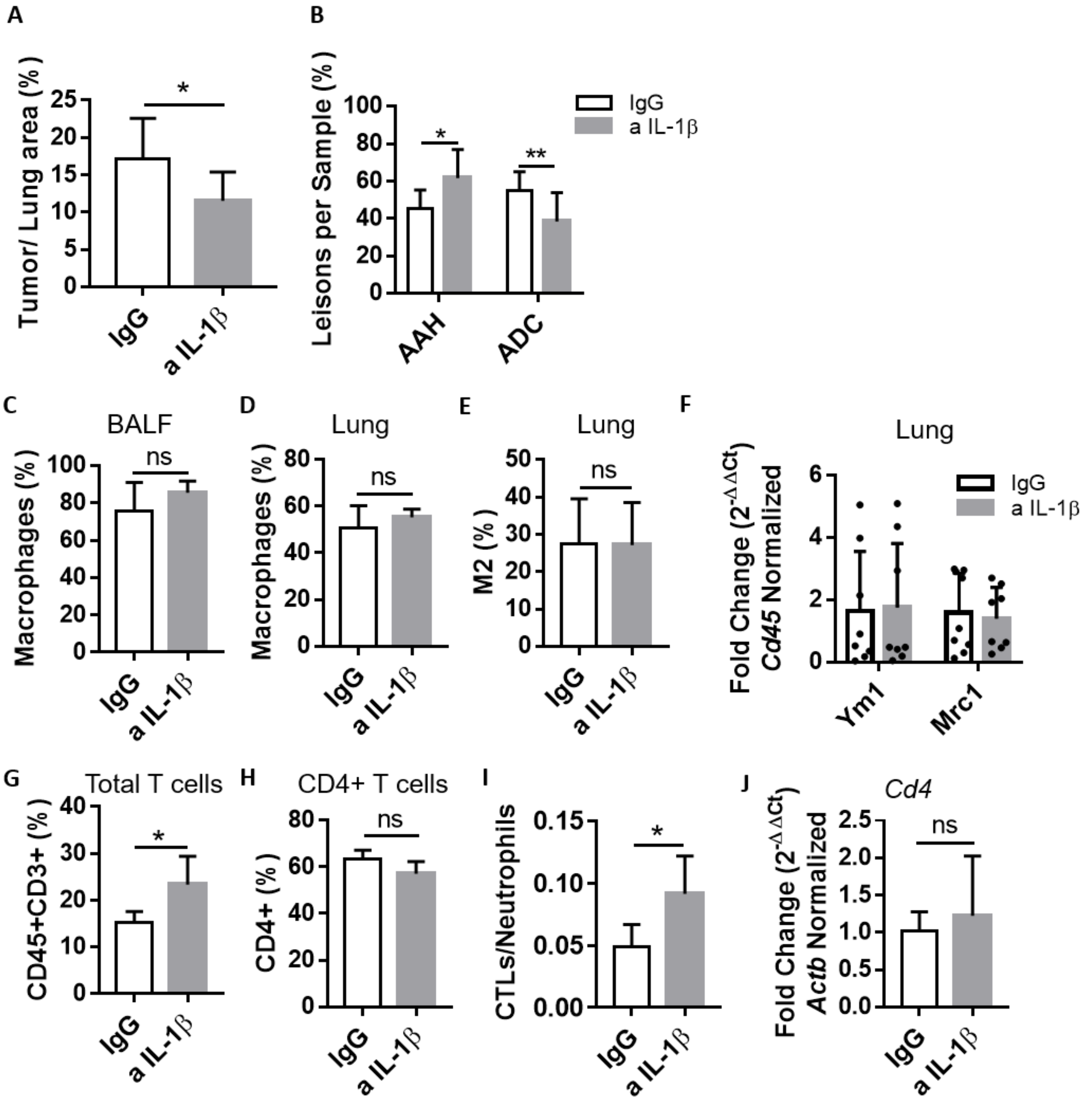

Supplementary Figure S1. (A) Tumor/Lung area percentage (n=7). (B) The proportion of atypical adenomatous hyperplasia (AAH) or adenoma/adenocarcinoma (ADC) lesions in each sample (n=7). Percentage of total macrophages in the (C) bronchoalveolar lavage fluid (BALF) and (D) the whole lung (n=3-4). (E) Percentage of M2 macrophages in the whole lung (n=3-4). (F) Relative mRNA expression of *Ym1*, *Mrc1* in the whole lung, normalized to *Cd45* expression (n=8-9). (G) Percentage of CD3+ T cells in the whole lung (n=3-4). (H) Percentage of CD4+ T cells in the whole lung (n=4-5). (I) The ratio of IFN-γ expressing CD8+ T cells (CTLs) to neutrophils among all the CD45 positive populations at 14-week-old mice (n=4-5). (J) Relative mRNA expression of *Cd4* in the whole lung, normalized to *Actb* expression (n=7-9). Data represent mean ± SEM; unpaired t-test, *p<0.5, **p<0.01.

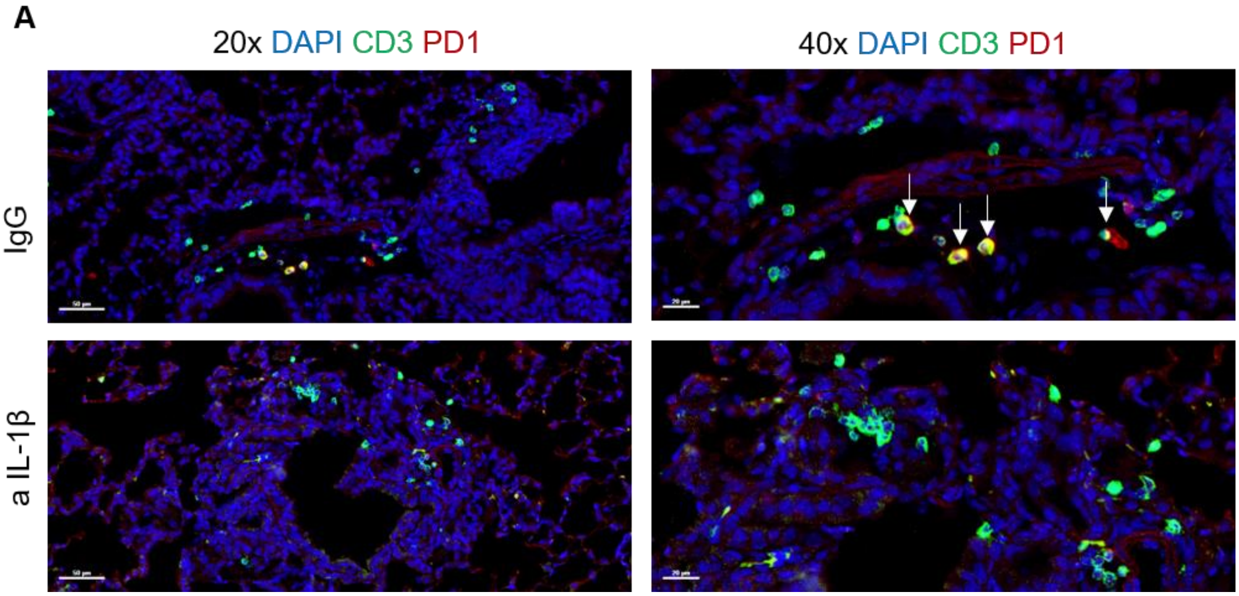

Supplementary Figure S2. (A) Fluorescent multiplex immunohistochemistry visualization of CD3 and PD-1 co-expressing cells (20x scale bar= 50μm; 40x scale bar= 20μm).

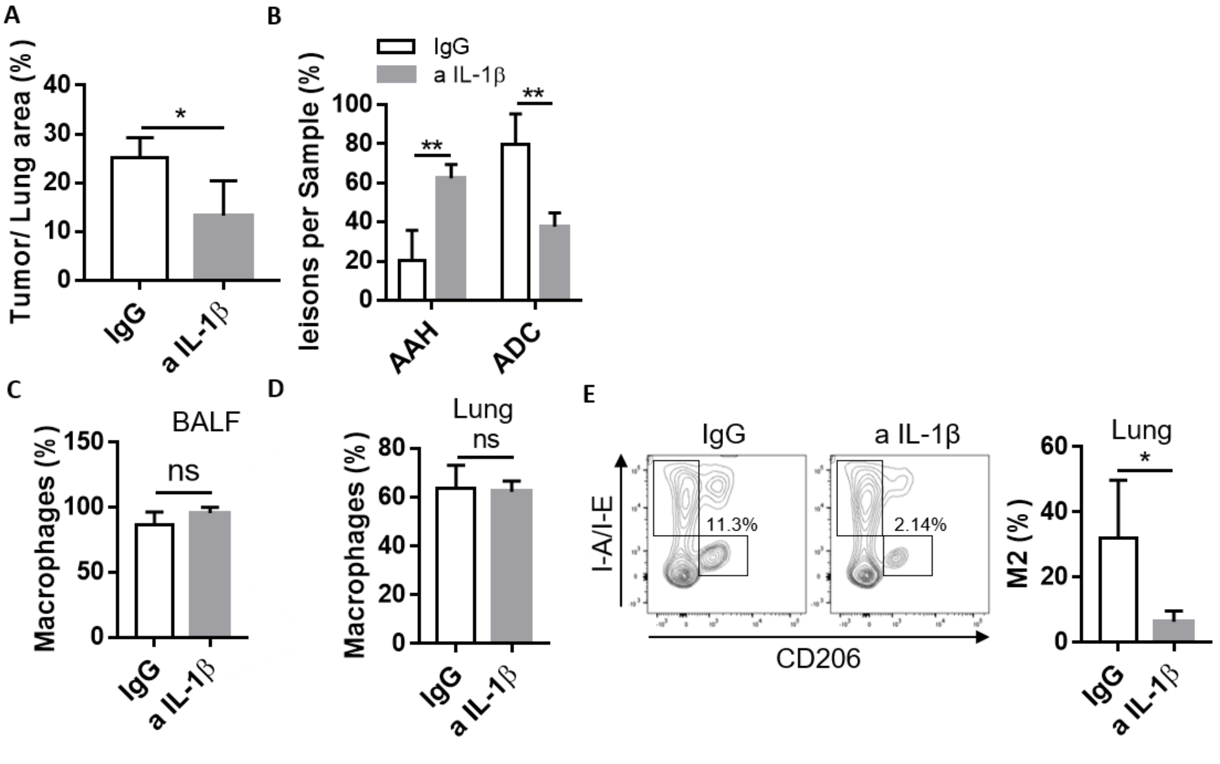

Supplementary Figure S3. (A) Tumor/Lung area percentage (n=4). (B) The proportion of AAH or adenoma/adenocarcinoma lesions in each sample (n= 4). Percentage of total macrophages in the (C) BALFs and (D) the whole lungs (n=3-6). (E) Percentage of M2 macrophages in the whole lungs (n=3-6).

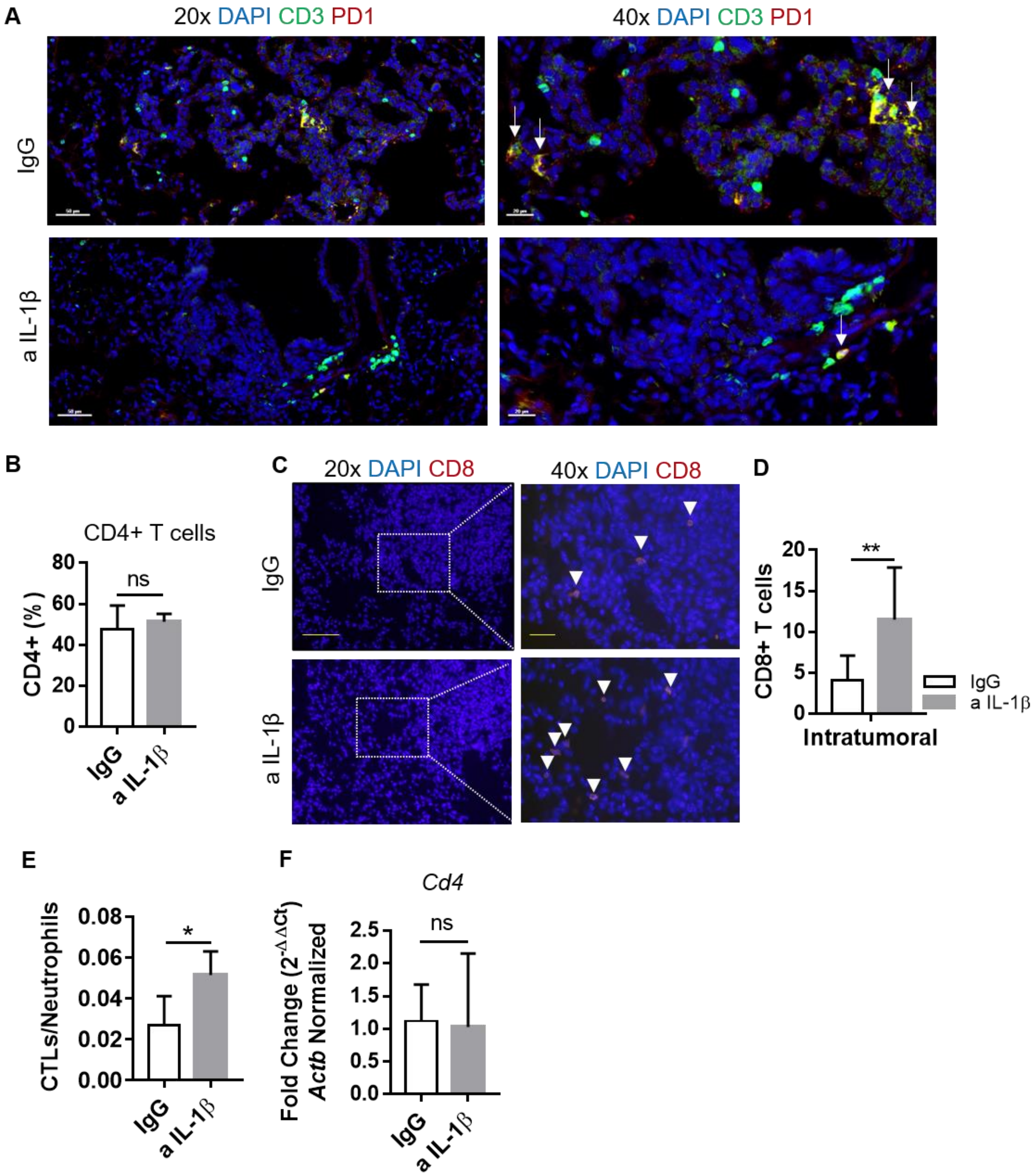

Supplementary Figure S4. (A) Fluorescent multiplex immunohistochemistry visualization of CD3 and PD-1 co-expressing cells (20x scale bar= 50μm; 40x scale bar= 20μm). (B) Percentage of CD4+ T cells in the whole lung (n=4-6). (C) Representative photomicrographs and (D) quantification of immunofluorescence staining with anti-CD8 (red) and DAPI (blue) in tumor lesions (20x scale bar= 50μm; 40x scale bar= 20μm) (n=10). (E) The ratio of CTLs to neutrophils among all the CD45 positive populations at 18-week-old mice (n=4-5). (F) Relative mRNA expression of *Cd4* in the whole lung, normalized to *Actb* expression (n= 4-5). Data represent mean ± SEM; unpaired t-test, ***p*<0.01, **p*<0.5.

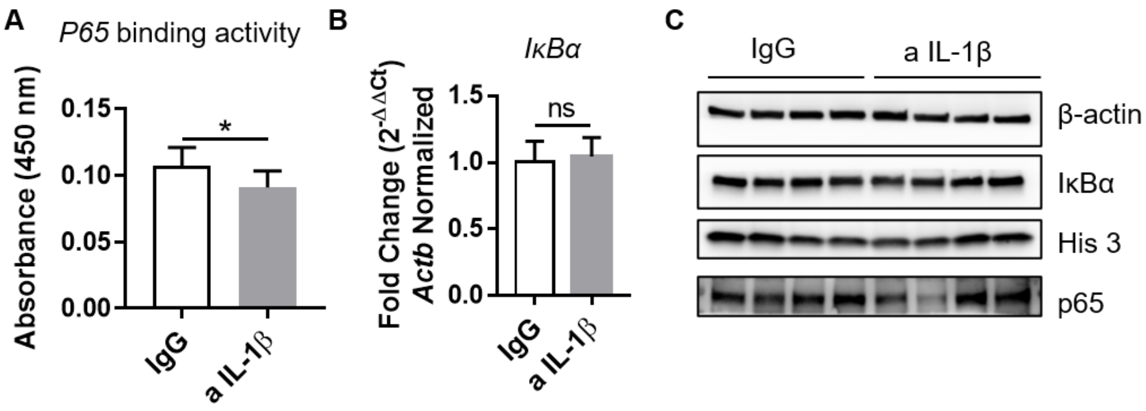

Supplementary Figure S5. (A) p65 binding activity of whole lung nuclear protein from 14-week-old CC-LR mice presented by OD value (n= 5-6). (B) Relative mRNA expression of *IκBα* in the whole lung of 18-week-old CC-LR mice, normalized to *Actb* expression (n= 4-5). (C) Western blot analysis of IκBα, β-actin protein levels in whole lung tissue and p65, Histone H3 (His H3) protein level in whole lung tissue nuclear extracts of 18-week-old CC-LR mice. Data represent mean ± SEM; unpaired t-test, ns: not significant.

A

Lymphocyte Gating Strategy

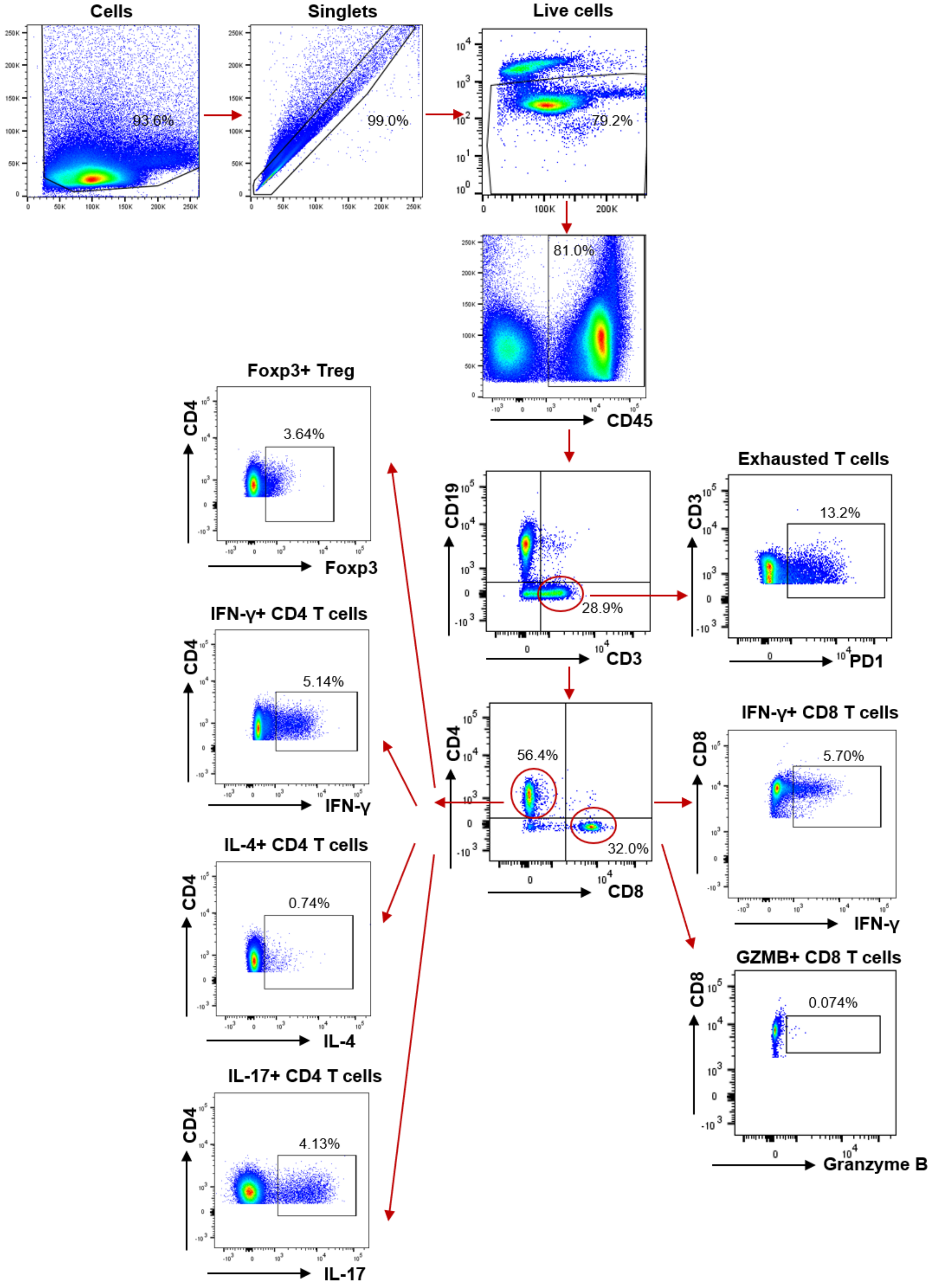

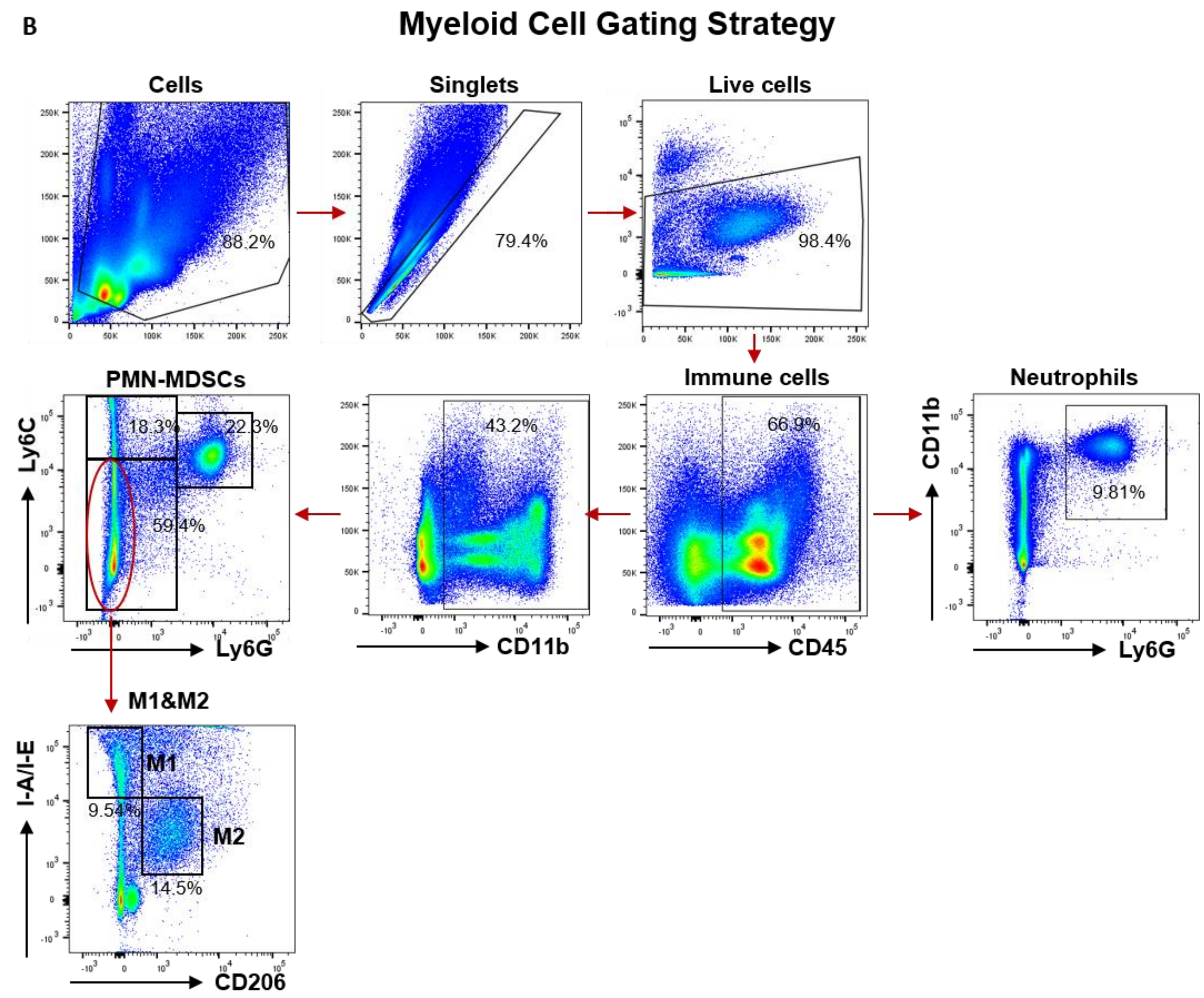

Supplementary Figure S6. Flow cytometry gating strategy for (A) lymphocytes and (B) myeloid cells.

Supplementary Table S1. List of antibodies used for the flow cytometry.

Flow cytometry			
**Lymphocyte Panel**			
**Antibody**	**Clone**	**Flourscence**	**Dilution Factor**
Ghost Dye™	--	Violet 510	1:150
CD45.2	104	redFluor 710	1:100
CD3	17A2	PerCP-Cy5.5	1:100
CD4	GK1.5	APC-Cy7	1:100
CD8a	53-6.7	PB	1:100
CD19	1D3	APC	1:100
IFN-γ	XMG1.2	FITC	1:100
IL-4	BVD6-2462	PE-Cy7	1:100
IL-17A	TC11-18H10	BV786	1:100
FoxP3	FJK-16S	PE	1:100
PD1	RMP1-30	BV711	1:100
Granzyme B	NGZB	PE-eFluor 610	1:100
**Myleoid Panel**			
**Antibody**	**Clone**	**Flourscence**	**Dilution Factor**
Ghost Dye™	--	Violet 510	1:100
CD45.2	104	redFluor 710	1:100
CD11b	M1/70	FITC	1:100
CD11c	N418	PE-Cy7	1:100
Ly6C	HK1.4	APC	1:100
Ly6G	1A8	PE	1:100
CD206	C068C2	BV421	1:100
I-A/I-E	M5/114.15.2	BV711	1:100

Supplementary Table S2. List of primers used in qRT-PCR.

Gene	Forward	Reverse
***Actb* (*β-actin*)**	5’GGCTGTATTCCCCTCCATCG3’	5’CCAGTTGGTAACAATGCCATGT3’
Cd45	5’ACCACCAGGTGAATGTCAATTT3’	5’CTTGCTTTCCCTCGGTTCTTT3’
Ym1	5'CAAGTTGAAGGCTCAGTGGCTC3'	5'CAAATCATTGTGTAAAGCTCCTCTC3'
***Mrc1* (*Cd206*)**	5'TTGGACGGATAGATGGAGGG3'	5'CCAGGCAGTTGAGGAGGTTC3'
Cd8a	5'CTGCGTGGCCCTTCTGCTGTCCT3'	5'GGGACATTTGCAAACACGCT3'
Cd4	5'GAAGATTCTGGGGCAGCATGGCAAAG3'	5'TTTGGAATCAAAACGATCAA3'
***Pdcd1* (*Pd1*)**	5'ACCCTGGTCATTCACTTGGG3'	5'CATTTGCTCCCTCTGACACTG3'
Il6	5’CTGATGCTGGTGACAACCAC3’	5’CAGACTTGCCATTGCACAAC3’
IκBα	5’CAGCAGACTCCACTCCACTT3’	5’GAGAGGGGTATTTCCTCGAA3’
